# A Fiber Optic Doppler Sensor and Its Application in Debonding Detection for Composite Structures

**DOI:** 10.3390/s100605975

**Published:** 2010-06-14

**Authors:** Fucai Li, Hideaki Murayama, Kazuro Kageyama, Guang Meng, Isamu Ohsawa, Takehiro Shirai

**Affiliations:** 1 State Key Laboratory of Mechanical System and Vibration, Shanghai Jiao Tong University, Shanghai 200240, China; E-Mail: gmeng@sjtu.edu.cn (G.M.); 2 Department of Systems Innovation, School of Engineering, The University of Tokyo, 7-3-1 Hongo, Bunkyo-ku, Tokyo 113-8656, Japan; E-Mails: murayama@giso.t.u-tokyo.ac.jp (H.M.); kageyama@giso.t.u-tokyo.ac.jp (K.K.); ohsawa@giso.t.u-tokyo.ac.jp (I.O.); shirai@giso.t.u-tokyo.ac.jp (T.S.)

**Keywords:** Fiber optic Doppler sensor, guided wave, debonding damage detection, composite structures

## Abstract

Debonding is one of the most important damage forms in fiber-reinforced composite structures. This work was devoted to the debonding damage detection of lap splice joints in carbon fiber reinforced plastic (CFRP) structures, which is based on guided ultrasonic wave signals captured by using fiber optic Doppler (FOD) sensor with spiral shape. Interferometers based on two types of laser sources, namely the He-Ne laser and the infrared semiconductor laser, are proposed and compared in this study for the purpose of measuring Doppler frequency shift of the FOD sensor. Locations of the FOD sensors are optimized based on mechanical characteristics of lap splice joint. The FOD sensors are subsequently used to detect the guided ultrasonic waves propagating in the CFRP structures. By taking advantage of signal processing approaches, features of the guided wave signals can be revealed. The results demonstrate that debonding in the lap splice joint results in arrival time delay of the first package in the guided wave signals, which can be the characteristic for debonding damage inspection and damage extent estimation.

## 1. Introduction

Composite materials are prime candidates for structural applications, such as aerospace, marine, automotive and infrastructure industries, because of their specific strength and stiffness but are expensive to maintain. The cost of inspection is approximately one third of the total cost of manufacturing and operating composite structures [1]. In order to realize their full potential, it is essential that they are maintained in a safe and economical manner.

A questionnaire based end-user requirements [2,3], performed under the Brite Euram project monitor, demonstrates that the major causes of in-service damage to composites structures in aerospace industry are low velocity impacts such as bird strike, runway stones and tool-drop (hangar rash) during maintenance and standby. These events lead to BVID (barely visible impact damage) which are prime sources of delamination and fiber cracking in composites structures. Statistics of the same questionnaire show also that debonding is considered as the second most important damage forms. This type of damage is linked to the dynamic loads submitted to the in-service structures or stress concentration.

Over recent years, a number of new technologies have evolved with the potential for automatic damage detection. Lamb wave detection technique is the most widely used guided-wave-based damage detection technique, *i.e.,* ultrasonic wave packets propagating in bounded media [4]. Although guided waves have demonstrated great potential for structural damage detection, to date, the practical commercial exploitation of ultrasonic guided waves has been very limited. This is related to several major drawbacks associated with the current guided-wave-based damage detection techniques, such as complex data interpretation and the requirement of baseline measurements, *i.e.*, data representing a ‘*no damage*’ condition (however these parameters can change due to temperature or bad coupling between the transducer and the structure).

Transducers play pivotal roles in guided-wave-based structural health monitoring (SHM) techniques. In the literature, a number of transducers have been applied. Piezoelectric (PZT) and fiber optic sensors are among the most attractive choices [5–10]. However, electromagnetic interference of PZT sensor sometimes limits its effectiveness in practical applications [11]. On the other hand, applications of fiber optic sensors are being quickly extended because of their flexibility, high strength, heat resistance, immunity to electromagnetic interference, and durability and corrosive resistance [12]. Hence, fiber-optic sensors are the most promising among all the currently developed sensors [5] for ultrasonic detection.

The major focus of interest among the fiber optic sensor community is fiber Bragg grating (FBG) sensor that has a series of parallel gratings printed onto the core of an optical fiber, and a narrow wavelength range of light is reflected from the sensors when a broadband light is illuminated [13–16]. Since the wavelength at the peak of the reflected signal is proportional to the grating period, the axial strain can be measured through the peak shift [5]. Further, the FBG sensor can be easily multiplexed. Therefore, a number of studies on guided wave detection using FBG sensors have been reported in the literature [5,11,13–16]. In the authors’ previous studies [17,18], a fiber optic Doppler (FOD) sensor, based on the Doppler effect of light wave transmission in optical fiber, was proposed and used in ultrasonic detection. Further, applications of the FBG and the FOD sensors in guided-wave-based SHM techniques were compared for the purpose of delaimnation detection in carbon fiber reinforced plastic (CFRP) laminates [18].

This work was devoted to debonding damage detection of lap splice joints in CFRP structures by using the FOD sensor. Firstly, principle of the FOD sensor is briefly introduced. Doppler frequency shift measurement using interferometers based on two types of laser sources, namely the He-Ne laser and the infrared semiconductor laser, are subsequently proposed and compared. Mechanics analyses of lap splice joint in CFRP structures are performed to optimize the locations of the FOD sensors, and a self-calibration FOD sensor network is accordingly constructed for debonding damage detection. In the experiments, piezoceramic disc and the FOD sensors are bonded on surface of CFRP structures with lap splice joint, functioning as actuator and sensor to generate and acquire guided ultrasonic waves, respectively. Debonding damages are assessed by using characteristics of guided wave signals.

## 2. Fiber Optic Doppler (FOD) Sensor

### 2.1. Principle of the FOD Sensor

The FOD sensor is based on the Doppler effect of light wave transmission in optical fiber. Consider the light wave, with frequency *f*_0_, transmission in an optical fiber with refractive index *n* and length *L*. When an accident causes the length of the fiber to change from *L* to *L* + *dL* in an infinitesimal time *dt*, the Doppler frequency shift _Δ_*f* can be obtained by [12,17]

(1) $${}_{\Delta }f=-\frac{n}{\lambda_{0}}\cdot \frac{\mathrm{dL}}{\mathrm{dt}}$$

where *λ*_0_ is the light wavelength in the vacuum, and *λ*_0_/*n* is the light wavelength in the media.

**Figure 1.**
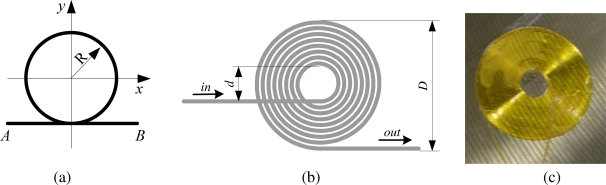
(a) The circular loop FOD sensor. (b) Sketch of the spiral FOD sensor. **(c)** Picture of a surface-bonded spiral FOD sensor.

In the previous study [17], three kinds of FOD sensors with different shapes were proposed, named circular loop FOD sensor, U-shape sensor, and elongated circular loop FOD sensors, respectively. Common shape of these FOD sensors is the circular part, as sketched in Figure 1(a). Points *A* and *B* denote the light source and observer, respectively. The theoretical Doppler frequency shift _Δ_*f* of the circular loop FOD sensors is obtained by [12,17]

(2) $${}_{\Delta }f=-\frac{\pi {\mathrm{Rn}}_{\mathrm{eq}}}{\lambda_{0}}({\dot{\varepsilon }}_{x}+{\dot{\varepsilon }}_{y})=-\frac{\pi {\mathrm{Dn}}_{\mathrm{eq}}}{2\lambda_{0}}({\dot{\varepsilon }}_{x}+{\dot{\varepsilon }}_{y})$$

where *ε̇_x_* and *ε̇_y_* are the strain rates on *x*- and *y*- axes, respectively, *R* and *D* are radius and diameter of the circular part of the FOD sensor, respectively, *n_eq_* is the equivalent refractive index of the waveguide and *λ*_0_/*n_eq_* is the equivalent length of light wave in the waveguide. If the effective sensing length of FOD sensor is defined as the total length in the sensing area (denoted by *L*), it is evident that the Doppler frequency shift _Δ_*f* is directly proportional to effective sensing optical fiber length of the FOD sensor.

In practical applications, the FOD sensor is usually fabricated into spiral shape, so as to make it easy to be bonded on surface of structure or embedded in the structure. Moreover the spiral shape can elongate the sensing optical fiber and therefore increase sensitivity of the FOD sensor. A spiral FOD sensor is schematically shown in Figure 1(b). Figure 1(c) depicts a surface-bonded spiral FOD sensor. Properties of the optical fiber used to fabricate the spiral FOD sensor in this study is listed in Table 1. Inner and outer diameters of the spiral FOD sensors applied in the present study are 8 mm and 21.2 mm, respectively.

### 2.2. Doppler Frequency Shift Measurement Systems

The Doppler frequency shift _Δ_*f* of the FOD sensor is measured using interference of two light waves with different frequencies. Therefore measurement instrument of the frequency shift for the FOD sensor was named interferometer. Light source of the interferometer should satisfy the following three requirements: (1) heterodyne, (2) high coherent ability, and (3) capability of emitting single-mode light waves. Based on these requirements, two types of interferometers are proposed and introduced in the present study, which are based on two kinds of light sources, viz. the visible He-Ne laser and the infrared semiconductor laser.

**Figure 2.**
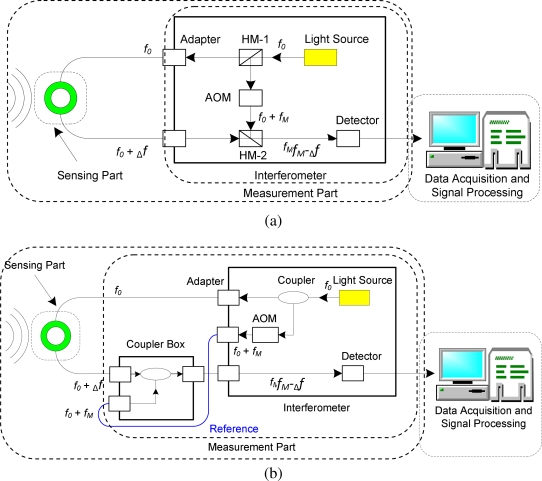
Principles of interferometers using (a) the He-Ne laser and (b) infrared semiconductor laser.

Principle of the He-Ne-laser-based interferometer is schematically shown in Figure 2(a), in which the light source is He-Ne laser (output power: 1 mW, wavelength *λ*_0_: 632.8 nm) and heterodyne interference technique is applied in the measurement. Firstly, the light source with stable frequency *f*_0_ is emitted into the upper half mirror (HM-1), which is diverged into two beams of light. Frequency of one beam of the light is changed from *f*_0_ to *f*_0_ + *f_M_* (*f_M_* = 80*MHz*) by an acousto-optic modulator (AOM). On the other hand, frequency of the light to the FOD sensor is changed from *f*_0_ to _Δ_ *f + f_0_* provided that there is optical fiber length change in the FOD sensor. Then, those two light beams interfere with each other and therefore beating signals with frequency of *f_M_* – _Δ_*f* (*f_M_* ≫ _Δ_*f*) are produced. Finally, a frequency-voltage convertor in the detector is used to offer voltage output for direct ultrasonic acquisition, as shown in Figure 2(a).

Hence, the larger the Doppler frequency shift _Δ_*f*, the higher the output voltage. According to Equation (2), when the strain rate in host structure is not high enough, the output voltage can be enlarged by elongating the effective sensing length of the FOD sensor at the sensing part in Figure 2(a). Therefore, sensitivity of the FOD sensor is relatively high in comparison with the FBG sensor, since the effective sensing optical fiber length of FOD sensor can be increased by using spiral shape [17,18].

For the convenience of description, the light transmitting from HM-1 to HM-2 through the FOD sensor is defined “signal light” and, on the other hand, the light transmitting from the HM-1 to the HM-2 through the AOM is defined “reference light”, as shown in Figure 2(a). It is evident that the waveguide length of the signal light can be different from that of the reference light, since the former can be variable in different applications by changing the outer and inner diameters of the sensor and, in contrast, the latter is fixed for a certain interferometer. In the case of the He-Ne-based interferometer, it is not necessary to consider this difference because the coherence length of the He-Ne laser is relatively long. However, the He-Ne laser has three intrinsic disadvantages, namely, (1) high propagation loss, (2) difficult to offer high power light with low cost, and (3) relatively low stability under long-term operation. These disadvantages make the He-Ne-laser-based interferometer only applicable in laboratory measurements, whereas, difficult to be widely used in practical engineering applications.

Based on the above mentioned drawbacks of the He-Ne-laser-based interferometer, the infrared-semiconductor-laser-based interferometer was further proposed to measure the Doppler shift of FOD sensor. Principle of this interferometer is sketched as Figure 2(b), in which wavelength of the infrared semiconductor laser is 1,550 nm. In this case, a reference waveguide is necessary, since the coherence length of the infrared semiconductor laser is relatively short. The reference waveguide is shown as the reference part (blue line) in Figure 2(b). To increase the signal-to-noise ratio (SNR) of measured signal, the waveguide length of the reference light should be as similar as possible to that of the signal light. The infrared-semiconductor-laser-based interferometer can overcome the above mentioned shortcomings of the He-Ne-laser-based interferometer.

### 2.3. Guided Waves Detection Using the FOD Sensor

Ultrasonic detection using the He-Ne-laser-based interferometer has been systematically investigated in the previous study [17]. In the present study, guided ultrasonic wave detection using the above mentioned two kinds of interferometers is further examined for the purpose of applications in debonding detection. Hanning-windowed tone-bust with 5 sinusoidal cycles was applied as incident wave, amplified and emitted into a piezoceramic disc bonded on surface of plate-like structure to excite guided waves propagating in the structure [19,20].

**Figure 3.**
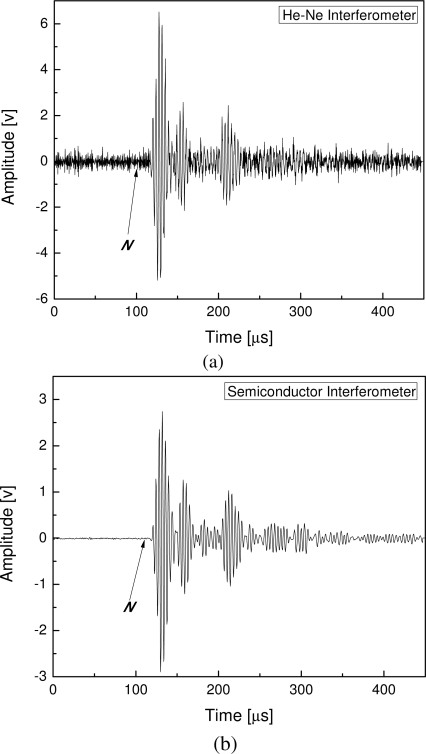
Original guided ultrasonic wave signals acquired using (a) the He-Ne-laser-based interferometer and (b) the infrared-semiconductor-laser-based interferometer.

For illustration, original guided wave signals acquired from a CFRP laminate using the He-Ne-laser-based interferometer (abbreviated to He-Ne interferometer) and infrared-semiconductor-laser-based interferometer (abbreviated to semiconductor interferometer) are shown in Figure 3(a,b), respectively. It is evident that noise energy in the He-Ne interferometer-based signal is much higher than that of the semiconductor interferometer-based signal. The SNR is usually one of the most important parameters indicating performance of a sensor. For guided ultrasonic wave detection using the FOD sensor addressed in the present study, SNR of the captured signal, *S*, can be estimated by *SNR_s_* = 20log(*V_s_*/*N_rms_*), where *V_s_* and *N_rms_* denote ultrasonic response amplitude and the root-mean-square value of signal appeared prior to ultrasonic response (around ‘*N*’ point in Figure 3), respectively. Characteristics of the He-Ne interferometer have been investigated in the previous study [17]. Therefore, only the semiconductor interferometer is examined in detail in the present study. As already mentioned, output of the semiconductor interferometer is influenced by the optical fiber length of the reference light. Hence, characteristic of the semiconductor interferometer in guided ultrasonic wave detection is investigated by changing optical fiber length of the reference light.

**Figure 4.**
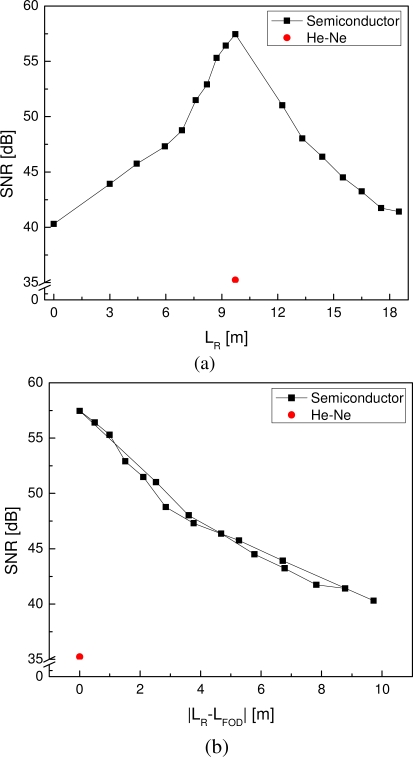
(a) SNR of guided wave signal changes with the optical fiber length of reference light of semiconductor interferometer. (b) SNR of guided wave signal changes with the absolute difference between optical fiber lengths of reference light and signal light.

Spiral FOD sensors with inner diameter 8 mm and outer diameter 21.2 mm were used to acquire guided ultrasonic wave signals. Optical fiber length of the signal light (including the sensing optical fiber of the FOD sensor and the free optical fiber) is about 9.72 m in the present study. Central frequency of the incident signal is 330 kHz and ultrasonic signals were acquired using sampling frequency 4 MHz. SNR results of the captured guided wave signals are shown in Figure 4, in which SNR of guided wave signal changing with optical fiber length of the reference light of the semiconductor interferometer is depicted in Figure 4(a) and SNR of guided wave signal changing with the absolute difference between optical fiber lengths of the reference light and the signal light is depicted in Figure 4(b).

In Figure 4(a), SNR reaches the maximum when the optical fiber length of the reference light is 9.73 m, which is almost equal to the optical fiber length of the signal light 9.72 m. However, SNR decreases at the both sides when optical fiber length of the reference light departs from that of the signal light in the figure. A further representation is proposed by using absolute value of the difference between the reference and signal optical fiber lengths, shown as the curve in Figure 4(b). It can be seen that the SNR curve section of shorter reference optical fiber matches very well with the SNR curve section of longer reference optical fiber, indicating that, in the case of semiconductor interferometer, reference with the same optical fiber length as the signal light (including the sensing optical fiber of the FOD sensor and the free optical fiber) offers the best SNR result in ultrasonic detection. Moreover, SNR of the guided wave signal captured using the He-Ne interferometer is about 35 dB in the current study, which is much smaller than all the semiconductor-interferometer-based results, as shown in Figure 4.

## 3. FOD Sensor Application in Debonding Assessment

Sixteen plies of Tenax-112 prepreg (Toho Tenax co., Ltd., Japan) were stacked in accordance with [0/90]_4s_. Cross-ply carbon fiber reinforced plastic (CFRP) laminates were fabricated and cut into beams with dimensions of *L* 245 mm × *W* 25 mm × *TH* 2.27 mm, to ensure identical properties for all CFRP beams. In practical applications, three kinds of joint methods have been applied to join different parts of a structure, namely, mechanical joints using bolts or rivets, weld using arc or spot welding, and joints using adhesives. In the present study, the adhesive method was used to join two pieces of the above-mentioned CFRP beams, to fabricate CFRP specimen with lap splice joints for the purpose of FOD-sensor-based debonding damage detection. Epoxy was selected as the adhesive. Properties of the CFRP beams and the epoxy applied in the present study are listed in Table 2.

**Figure 5.**
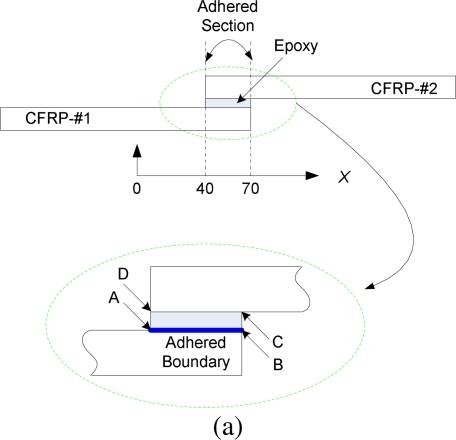

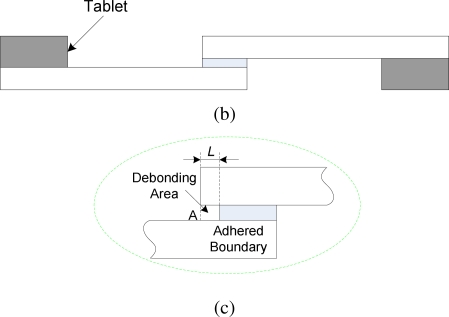
(a) Sketch of the specimen and zoom-in view of the lap splice joint. (b) Sketch of the specimen with tablets at the two ends in the simulation. (c) Zoom-in view of the lap splice joint with debonding.

To fabricate the specimen with lap splice joint, firstly, two of the above-mentioned CFRP beams were joined by using epoxy Araldite^®^ Adhesive 2011 (Huntsman International LLC.) and fastened with a clamp. Curing process was subsequently implemented, comprising two steps: 1) curing at room temperature for 24 hours with the clamp; 2) post curing at 40 °C for 16 hours without the clamp. Sketch of the specimen is shown in Figure 5(a). Length of the joint section and thickness of the epoxy layer are 30 mm and 0.15 mm, respectively. Moreover, two kinds of CFRP specimens were fabricated in this study, *viz.* intact specimen (completely bonded condition at the lap splice joint area) and specimen with debonding at the adhered section. Debonding was introduced by inserting thin Teflon^®^ films in the lap splice joint. Detail description of the debonding is in Section 3.1.

### 3.1. Elastic Analysis of Lap Splice Joint in CFRP Structure

**Figure 6.**
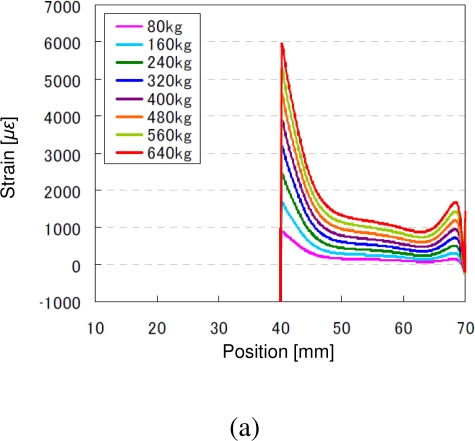

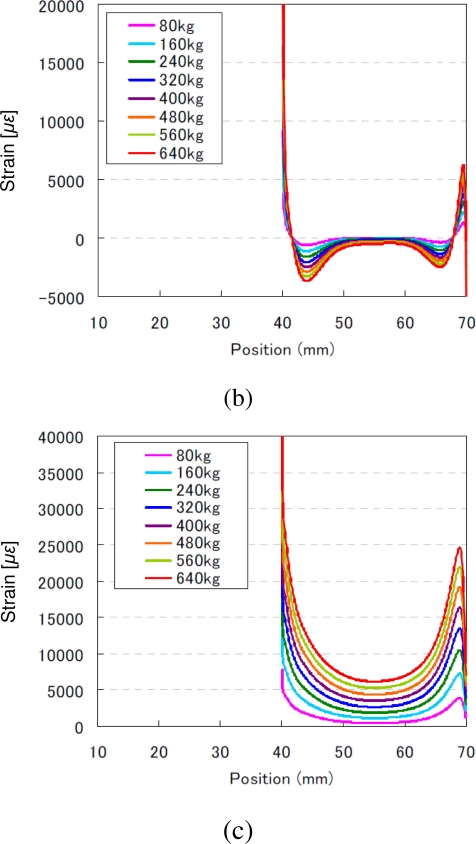
Strain distribution of the epoxy side at the adhered boundary in (a) longitude, (b) peel and (c) shear directions.

To investigate the characteristics of the lap splice joints in the CFRP structures, finite element analyses (FEA) were performed on the MSC.Marc Mentat (MSC.Software Corporation) software platform to simulate and complete elastic analysis for the specimens. Further, the transducer network can also be optimized based on the simulation results for the purpose of damage assessment. In the simulation, extension forces were added at the two ends of the specimen, which are changed from 0 kgf (kilogram-force) to 640 kgf with the step of 80 kgf. To minimize the torque effect on the lap splice joints, two tablets was added at the both ends of the specimen, as shown in Figure 5(b). Without losing generality, only part of the specimen, including the lap splice joint, was used to illustrate the simulation results, shown the *x*-axis in Figure 5(a). Simulation results are shown in Figure 6, in which strain distributions of the adhered boundary [as shown in Figure 5(a)] on the epoxy side were addressed in three directions, namely, longitude, peel and shear directions.

According to the simulation results, in the cases of all the three directions, maximum strain happens at the left end [point *A* in Figure 5(a)] of the joint section. Moreover, strain at the right end of the joint section is also relatively larger than that of the middle adhered section. These simulation results disclose that, in the case of lap splice joint condition addressed in this study, the strain distributions in all directions at the joint section have a concave shape, implying that debonding damage usually happens first from the ends [*A*, *B*, *C* and *D* in the zoom-in view in Figure 5(a)] of the joint section. Therefore, in the specimen fabrication process, debonding damages were introduced by inserting thin Teflon^®^ films in the lap splice joint. Debonding lengths, *L*, was measured from point *A* [as shown in Figure 5(c)], and were 0 mm (for the intact case), 5 mm, 15 mm and 25 mm in the experiments.

### 3.2. Transducer Network Setup

**Figure 7.**
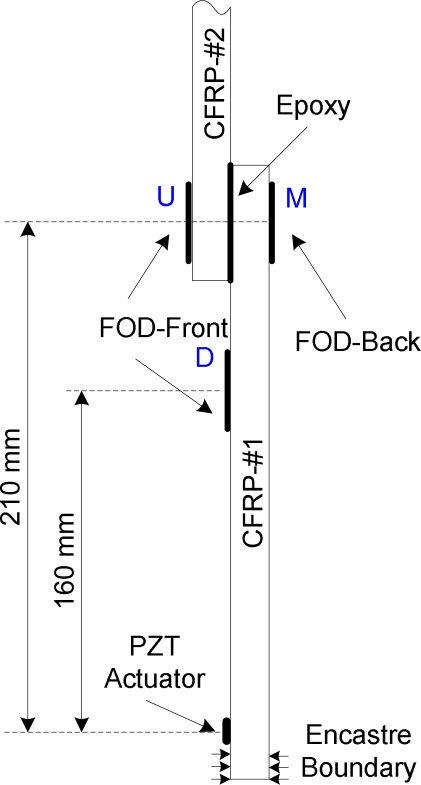
Transducer network setup in the experiments.

In guided-wave-based SHM system, at least one actuator is usually necessary to excite guided waves propagating in the host structures. In this study, a piece of piezoelectric (PZT) disc (*D* 6.9 mm × *TH 0*.5 mm) was bonded on surface of each CFRP specimen, functioning as the actuator of the SHM system. The spiral FOD sensor was used to acquire guided wave signals for debonding damage detection. The CFRP structures were fixed at one end and the actuator was bonded close to the encastra boundary of the structure, as shown in Figure 7.

The CFRP structures consist of three parts, viz. CFRP-#1, CFRP-#2 and the lap splice joint, as shown in Figure 7. It is evident that when guided waves are generated by the PZT actuator, they will propagate along the CFRP-#1 and then to the lap splice joint. Since the joint combine the CFRP-#1 and CFRP-#2 into one structure, the joint section will function as a medium or an actuator for the CFRP-#2. Therefore the guided waves will subsequently propagate in CFRP-#2. For the structures in this study, the FOD sensor can be bonded on the CFRP-#1, the lap splice joint or the CFRP-#2. However, for the CFRP-#2, as the joint just functions as a medium or an actuator, its size change usually cannot greatly influence the excited guided waves. Moreover, the joint can also result in the guided waves much more complicated, making it difficult for signal interpretation and damage detection. Therefore, it is not the optimal selection to capture guided wave signals on the CFRP-#2 for damage assessment. On the other hand, signal components reflected from the joint can be used for debonding damage detection if the guided wave signals are captured on the CFRP-#1. However, multiple modes and dispersion of guided waves are impediments in this case. Therefore, the FOD sensors were bonded on the joint area of the CFRP structures in this study. Moreover, one more FOD sensor was also bonded on surface of the CFRP-#1, in which only transmitted guided wave was used as baseline (reference) for damage detection.

The setup of the FOD sensors is shown in Figure 7, in which three spiral FOD sensors are used, named *U*, *M* and *D*, respectively. As already mentioned, baseline measurements, *i.e.*, data representing a ‘*no damage*’ condition, are usually required in guided-wave-based SHM techniques [4]. However, these parameters can change due to variable working conditions or bad coupling between the actuator and the structure. For the transducer network in the present study, both the baseline measurements and the current working condition can be measured at one excitation by using difference sensors, which is named self-calibration sensor network. For the sensor network shown in Figure 7, guided wave signals acquired by the sensors *M* and *D* function as baseline measurements and, on the other hand, those acquired by the sensor *U* function as signal for debonding damage detection. Moreover, the sensors *U* and *M* are located the same distance (210 mm) from the PZT actuator.

### 3.3. Debonding Damage Detection Using FOD Sensors

Signal processing approaches are usually required in guided-wave-based defect identification, since various affects, such as noise and dispersion of guided waves, usually result in the acquired ultrasonic signals much more complicated. In the previous study [18,20], a signal processing algorithm, based on finite impulse response (FIR) filter and Hilbert transform (HT), was proposed for the purpose of guided wave signal feature extraction. Envelopes of wave signals were consequently obtained to identify delamination defects in CFRP structures. The algorithm is successively applied to purify guided wave signals in this study.

**Figure 8.**
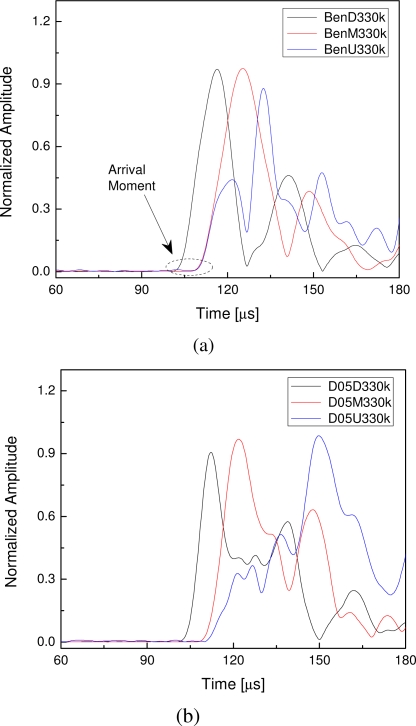

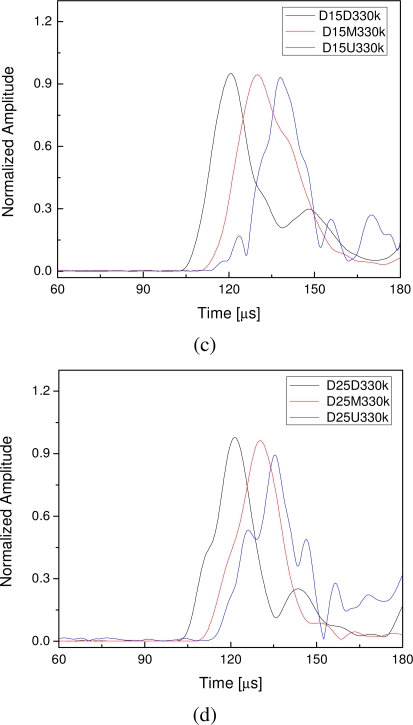
Normalized envelopes of guided wave signals captured using FOD sensors from (a) intact CFRP specimen and defected CFRP specimens with the debonding lengths (b) 5mm, (c) 15 mm and (d) 25 mm.

As aforementioned, two kinds of specimens were fabricated in this study, namely the intact and debonding CFRP structures with lap splice joints. Amplitude features of the guided wave signals are not considered, as excitation situation of the PZT actuator on the four specimens may be different because of different bonding situation. Therefore, amplitudes of the envelopes were normalized in the signal processing. Central frequency of the incident wave and sampling frequency of the signal are 330 kHz and 4 MHz, respectively. Envelopes of the FOD-sensor-captured signals with normalized amplitudes are shown in Figure 8. Guided wave signals in this section are measured by using the semiconductor interferometer. In the legends of Figure 8, ‘BenΔ330k’ denotes the result of intact specimen (benchmark) using the sensor ‘Δ’ (*D*, *M* and *U*), and, on the other hand, ‘D** Δ330k’ denotes the results of defected specimen with debonding length ‘**’ (5 mm, 15 mm and 25 mm) using the sensor ‘Δ’ (*D*, *M* and *U*). To make it much clearer, only a part of each sampled signal was shown in the figures and, moreover, arrival time of the incident signal is 75 μs in the experiments.

According to the results of the intact specimen in Figure 8(a), it is evident that several wave packages are present in wave signals, due to the multiple modes of guided waves and reflection from different discontinuities (such as lap splice joint and specimen ends). To avoid complicated signal interpretation and identification, in this study, only the first wave package of each signal is addressed for damage assessment. For the envelopes in Figure 8(a), the most evident characteristic is the arrival moment of each envelope, shown as the dashed-line circled area. Starting sections of the first wave packages of the signals captured by sensors *M* and *U* superpose with each other, illustrating arrival times of the two signals are same because of the locations of the sensors *M* and *U*, as shown in Figure 7. Group velocity of the first wave package can be estimated by using the arrival moments of the first envelopes in Figure 8(a) and the distances between the actuator and the sensors, which is circa 5.93 mm·μs^−1^.

For the CFRP structures with debonding in the lap splice joints, arrival moments of signals captured by sensors *D* and *M* remain invariable. In contrast, the first wave packages of the signals captured by sensors *U* arrive a little later than those captured by sensors *M*, as shown in Figure 8(b–d), which is caused by the debondings. The most evident characteristic is that the first wave packages of signals acquired by sensors *M* and *U* do not superpose with each other like the intact case in Figure 8(a). As already mentioned in Section 3.2, the lap splice joint of the CFRP structure serves as a medium transmitting the guided waves from CFRP-#1 to CFRP-#2 and the transmission starts when guided waves arrive point *A*, as shown in Figure 5(a). When debonding happens in the lap splice joint, as shown in Figure 5(c), the transmission will happen a little later. Hence, guided waves will arrive a little later in the *U*-captured ultrasonic signals provided that debonding happens. This time delay feature of the first wave package in the envelope of the *U*-sensor-based guided wave signal can be applied for damage identification. Both the signals captured by sensors *D* and *M* can be the reference signals to calibrate the arrival time of the *U*-sensor-based signal. Moreover, it is evident that it is much more distinct by using the *M*-sensor-based signal than by using the *D*-sensor-based signal, as shown in Figures 8(b), (c) and (d).

The above results illustrate that both the baseline and damage-related measurements can be completed in only one experiment. A sensing setup with this characteristic is defined as a self-calibration sensor network. In the most widely used guided-wave-based SHM techniques [4], baseline measurements (maybe tested a certain long time ago), *i.e.*, data representing a ‘*no damage*’ condition, are usually required, in which working conditions of the baseline measurements, such as circumstances (e.g., temperature, humidity and surrounding noise) and coupling condition of the actuator, may differ from the working conditions of the current measurement for damage detection. In contrast, this drawback can be overcome by using the self-calibration sensor network proposed in this study, since both the baseline (reference) and the damage-related signal are measured using one excitation at the same time.

**Figure 9.**
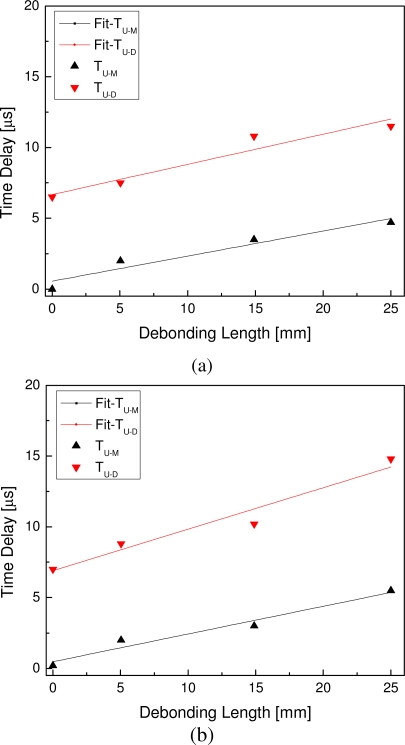
The relations and their linear fittings between the debonding length and the time delay at incident frequencies (a) 330 kHz and (b) 280 kHz.

Further, under the excitation frequency 330 kHz, the relations and their linear fittings between the debonding length and the time delay are shown in Figure 9(a), in which T_U-M_ and T_U-D_ denote the time delay between the sensors *U* and *M* and that between the sensors *U* and *D*, respectively. It reveals that the time delay is directly proportional to the debonding length. Considering the group velocity of the first wave package in the guided wave signals 5.93 mm·*μ*s^−1^, debonding lengths are further estimated and listed in Table 3. Moreover, for verification, experiments were subsequently conducted under different incident frequency 280 kHz with the results shown in Figure 9(b), demonstrating that similar conclusions can be obtained.

## 4. Discussion

**Figure 10.**
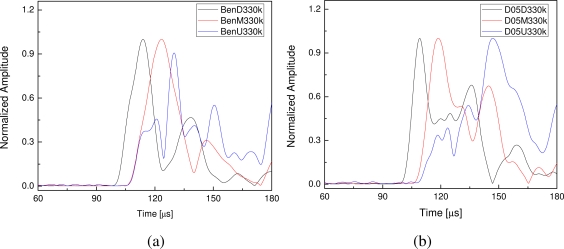
Normalized envelopes of guided wave signals measured by using He-Ne-based interferometer from (a) intact specimen and (b) defected specimen with debonding lengths 5 mm.

For comparison, the He-Ne interferometer was further used to measure the guided wave signals from the specimens conducted in the present study. Based on the extracted features of the acquired guided wave signals, the SNRs of the signals captured by the sensors *D* and *M* are acceptable for all the four specimens. Moreover, we could also acquire usable signals acquired by the sensors *U* on the intact and the 5 mm-debonding specimens. However, noise on *U*-sensors-based wave signals of the 15 mm-bebonding and 25 mm-debonding specimens are relatively so strong that those signal was not capable of inspecting the debonding damages, which is because of the aforementioned shortcomings of the He-Ne interferometer and complicated wave transmission mechanism of the lap splice joint in the CFRP structures. The results obtained by using measurements of the He-Ne interferometer are shown in Figure 10, in which only the intact and 5 mm-debonding cases are shown as the 15 mm- and 25 mm-debonding cases are not available. These results verify the Doppler frequency shift measurement comparison between the He-Ne and the semiconductor interferometers investigated in Section 2.

Overall size of the specimens used in the present study is quite small in comparison with those of typical composite structures employed in the aforementioned aerospace and other industries. Generally speaking, the size of the bonding area in composite structures is relatively small compared to the overall size of the structures. Therefore, in practical applications, the proposed self-calibration sensor network can be distributed around the local bonding area, instead of the whole structures, for debonding damage detection. Furthermore, in the literature, both fiber-optic and piezoelectric sensors have been embedded in composites to monitor health status of the in-service structures, as well as protecting transducers from damages. Thickness of the proposed FOD sensor is the same as FBG sensor, namely diameter of the optical fiber, and quite smaller than most of piezoelectric sensors. Hence, the FOD sensors can also be embedded in composite structures, instead of mounted on surface of structure, to protect them from damage in practical applications.

## 5. Conclusions

In this study, both the He-Ne-laser-based and the infrared-semiconductor-laser-based interferometers were proposed for the purpose of guided wave detection using fiber optic Doppler (FOD) sensor. The infrared semiconductor laser can overcome drawbacks of the He-Ne laser, such as high propagation loss, high cost and low long-term stability. Therefore, the semiconductor interferometer could offer higher signal-to-noise ratio in FOD sensor-based ultrasonic detection, provided that optical fiber length of the reference light is equal to that of the signal light. The proposed FOD sensor measurement systems were further used in debonding damage identification in carbon fiber reinforced plastic (CFRP) structures with lap splice joints. Based on elastic analysis of the structure, a self-calibration FOD sensor network was constructed to overcome the shortcomings of the conventional sensor network in guided-wave-based structural health monitoring techniques, in which both the baseline (reference) and the signal containing damage information can be measured by only one excitation. The results demonstrate that debonding causes time delay between the baseline and the damage-related signal. Moreover, the time delay is direct proportional to the debonding length in the lap splice joints.

## Figures and Tables

**Table 1.** Properties of the optical fiber used to fabricate the FOD sensor.

**Product Name**	**Heat-Resistant Optical Fiber**
MFD (*μ*m)	10.9 (wavelength 1,550 nm)
	9.5 (wavelength 1,310 nm)
Clad diameter (*μ*m)	125
Outer diameter of finished product (*μ*m)	150
Transmission loss	0.5 dB/km or less (wavelength 1,310 nm, 1,550 nm)
Sheath materials	Polyimide resin
Allowable temperature limit (°C)	300 (Short period)
	200 (Long period)
Tensile failure strength (N)	50 (room temperature)
Minimum bending diameter *Φ* (mm)	5

**Table 2.** Properties of the CFRP laminate and epoxy.

**Materials**	**Young’s Modulus (GPa)**	**Poisson’s ratio**	**Density (kg/m^3^)**
CFRP	84.8	0.043	1550
Epoxy	3.6	0.43	1150

**Table 3.** Time delay and estimated debonding length.

**Specimen**	**T_U-M_ (*μ*s)**	**T_U-D_ (*μ*s)**	**Debonding Length (mm)**
Intact	0	6.5	0
D05	2.0	7.5	11.9
D15	3.5	10.8	20.8
D25	4.7	11.5	27.9

Note: The ‘D**’ denotes defected specimen with debonding length ‘**’
